# Antioxidant Status of Cyanobacteria Strains During Long-Term Cultivation in Nitrogen-Free Media

**DOI:** 10.3390/ijms262210891

**Published:** 2025-11-10

**Authors:** Irina Maltseva, Aleksandr Yakoviichuk, Svetlana Maltseva, Maxim Kulikovskiy, Yevhen Maltsev

**Affiliations:** 1 Faculty of Natural Sciences, Melitopol State University, 20 Lenina St., 72312 Melitopol; maltseva-irina22@yandex.ru (I.M.); alex.yakov1991@gmail.com (A.Y.); 2 K.A. Timiryazev Institute of Plant Physiology RAS, IPP RAS, 35 Botanicheskaya St., 127276 Moscow, Russia; svetadm32@gmail.com (S.M.);

**Keywords:** α-tocopherol, enzymes, fatty acids, protein, retinol, thiobarbituric acid reactive substances

## Abstract

This study examines the antioxidant status of four *Nostoc* and *Desmonostoc* strains during long-term cultivation in nitrogen-depleted media. Growth rates, retinol and α-tocopherol content, fatty acid composition, and activities of antioxidant enzymes were analysed. The results showed that all tested strains adapted to nitrogen limitation using various cellular mechanisms. Specifically, the strain *Nostoc sphaeroides* exhibited the highest specific growth rate and elevated glutathione peroxidase activity. The *Nostoc commune* and *Desmonostoc caucasicum* strains displayed higher superoxide dismutase activity, suggesting robust antioxidative capabilities. Additionally, *Desmonostoc caucasicum* exhibited unique adaptive strategies, such as elevated succinate dehydrogenase activity. Generally, fatty acid composition changes showed divergent lipid peroxidation vulnerabilities among the studied strains. Principal component analysis highlighted clear distinctions among the strains in terms of their antioxidant capacities and metabolic adjustments. High retinol content correlated positively with increased catalase activity and fatty acid saturation, whereas α-tocopherol concentration was linked to succinate dehydrogenase activity. The obtained results underscore the robustness of cyanobacterial antioxidant defence systems and highlight their metabolic adaptations under nitrogen deprivation. Understanding these responses offers insight into potential biotechnological applications, such as biofertilizers or therapeutics.

## 1. Introduction

Achieving the Sustainable Development Goals is a priority in the present day. The solution to this problem largely depends on harnessing the potential of living organisms, which have virtually unlimited metabolic capabilities and a variety of ecological possibilities that scientists have yet to explore.

Cyanobacteria are some of the most abundant photosynthetic microorganisms. They have unique cellular strategies to adapt to life in different environments. Cyanobacteria live in freshwater and saline reservoirs, on the surface and in the deep horizons of soil, on rock substrates, on tree bark, etc. [1]. Cyanobacteria can survive in extreme conditions of various factors (temperature, light, salinity, etc.). They are among the first to settle on technogenic substrates and initiate the primary soil formation processes [2]. In arid regions, they, together with eukaryotic microalgae, lichens and mosses, are part of the biological soil crust (BSC). BSCs perform critical ecological functions, such as soil stabilisation and nutrient enrichment [3,4]. Cyanobacteria and eukaryotic microalgae form the basis of food chains in aquatic ecosystems. However, the massive growth of cyanobacteria in aquatic ecosystems, which has become more frequent due to climate change, threatens aquatic organisms and human health. Some cyanobacteria can synthesise cyanotoxins: microcystin, nodularin, anatoxin, and saxitoxin [5]. It is cyanobacterial bloom that is particularly harmful. Toxins can affect specific organs in humans and animals, leading to various health issues. However, these and other biomolecules synthesised by cyanobacteria have great pharmacological importance. Medicines based on them have antibacterial, antifungal, and antitumor effects [6].

Cyanobacteria, like eukaryotic microalgae, are a valuable source of natural compounds. They can accumulate significant amounts of proteins, carbohydrates, lipids, fatty acids (FAs), pigments, vitamins, and other compounds. These compounds are in high demand in various industries, such as the cosmetic and pharmaceutical sectors, as well as in the production of agricultural products, aquaculture, biofuels, and various food and additives [7,8].

Recently, there has been increasing interest in cyanobacteria and their ability to stimulate crop growth, reduce crop stress, increase soil fertility through carbon fixation, and fix nitrogen [9,10].

Biological nitrogen fixation is the largest natural source of new nitrogen for most terrestrial ecosystems [11]. Many cyanobacteria, primarily representatives of heterocytes, can fix atmospheric nitrogen. They convert atmospheric N_2_ into biologically available forms. At the same time, they ensure their own growth and make nitrogen available to other living organisms [10].

Species from the genus *Nostoc* Vaucher ex Bornet et Flahault inhabit various aquatic and terrestrial habitats. They have symmetrical trichomes that are located in massive mucous colonies. *Nostoc* cells are devoid of gas vacuoles and are usually very constricted at the transverse partitions. *Nostoc* has specialised heterocyte cells that allow it to fix N_2_. To date, from the genus *Nostoc*, based on a combination of molecular, morphological, and ecological criteria, Řehaková et al. [12] has isolated the genus *Mojavia* K. Reháková et J. R. Johansen, and Hrouzek et al. [13] has isolated the genus *Desmonostoc* Hrouzek et S.Ventura.

Numerous studies have focused on the analysis of *Nostoc*-like organisms for the production of various biotechnologically valuable metabolites, as well as their use as biofertilizers in agricultural systems [10,14]. Several studies have shown that the use of various stress factors (lighting, nitrogen and phosphorus starvation, etc.) changes the productivity of lipids, proteins, and other valuable compounds [15,16,17]. Poveda [18] noted that cyanobacterial metabolites stimulate crop growth and increase plant resistance to various abiotic stresses. This effect is possible due to the direct impact of cyanobacteria on the soil or through the activation of plant responses [18].

It is known that stress causes metabolic disorders in cells and disrupts the course of many biosynthetic pathways. In the case of metabolic imbalance, both radical and non-radical reactive oxygen species (ROS) accumulate in cells. At physiological concentrations, they play a vital role in cell signalling. However, during stress, excessive amounts of ROS disrupt the redox state of cells. ROS can oxidise and damage critical cellular molecules, such as proteins, lipids, and DNA, accumulating harmful by-products. As a result, ROS and by-products damage cell membranes, disrupting catabolic and anabolic processes. Large-scale damage can lead to cell death [19]. To control ROS production, cells have enzymatic (superoxide dismutase (SOD), catalase (CAT), peroxidase (PO), glutathione peroxidase (GPx), etc.) and non-enzymatic antioxidant defence systems (AOSs) (glutathione, tocopherols, ascorbic acid, carotenoids, anthocyanins, etc.). These components help to inactivate free radicals (including ROS), reduce their number or prevent their formation, and protect cells from oxidative damage [20].

It should be noted that the antioxidant protection of cyanobacteria has not yet been well described. To date, several scientists have conducted studies on the antioxidant protection of cyanobacteria. These studies demonstrate changes in enzymatic and non-enzymatic antioxidant levels after exposure to Cu^2+^, salinity, high temperature, and varying light intensity [21,22,23]. It is necessary to continue researching the antioxidant protection system of cyanobacteria, especially under nutritional stress conditions. Studies of the antioxidant protection system of cyanobacteria under food stress are essential for solving various biotechnological tasks, including restoring soil fertility and enriching it with nitrogen compounds, as well as enhancing the stability of crops and increasing their antioxidant status.

When studying the diversity and ecology of cyanobacteria and microalgae in soils from various ecosystems, researchers have repeatedly noted representatives with morphological features that correspond to *Nostoc sensu lato*. They often have a dominant role in cyanobacteria and microalgae soil communities [24,25]. Due to their ability to fix carbon and nitrogen, they are essential in forming primary products. We isolated some samples into cultures and identified them based on morphological and phylogenetic analysis.

The purpose of this work was to study the interactions underlying the maintenance of cellular homeostasis in four strains of cyanobacteria and the coordination of reactions during growth under conditions of nitrogen starvation, using the example of changes in the content of retinol (vitamin A) and α-tocopherol (vitamin E) (low-molecular-weight components of AOSs), as well as the activity of AOS enzymes.

## 2. Results

### 2.1. Molecular Analyses

**Figure 1:**
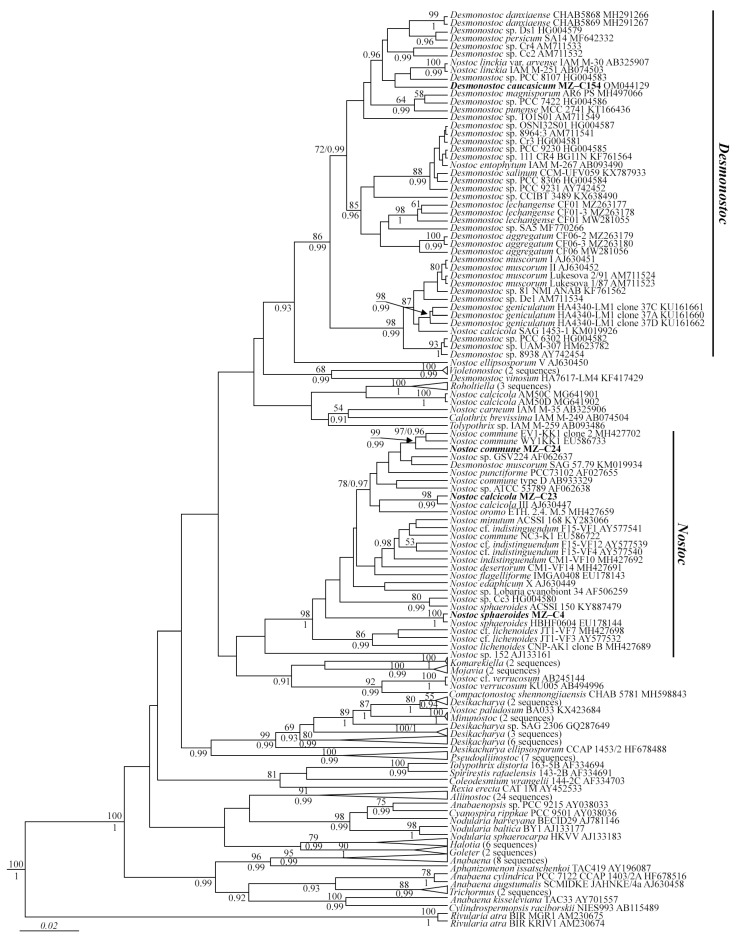
Phylogenetic position of new *Nostoc* and *Desmonostoc* strains (indicated in bold) within the Nostocales, based on Bayesian inference for the partial 16S rRNA gene. The total length of the alignment is 1607 characters. Values above the horizontal lines are bootstrap proportions (>50%) from ML analyses; values below the horizontal lines (or to the right of the slash) are Bayesian posterior probabilities (>0.9). Strain numbers (if available) and GenBank accession numbers are indicated for all sequences.

To evaluate the phylogenetic position of the studied strains, we sequenced the ribosomal 16S rRNA gene. The strains *Nostoc sphaeroides* Kützing ex Bornet et Flahaul MZ–C4, *Nostoc calcicola* Brébisson ex Bornet et Flahault MZ–C23, and *Nostoc commune* Vaucher ex Bornet et Flahault MZ–C24, together with other *Nostoc sensu stricto* species, were grouped into a unique cluster with high statistical support: likelihood bootstrap (LB), 98%; posterior probability (PP), 1.0 (Figure 1). *N*. *calcicola* MZ–C23 was most similar to the strain *N*. *calcicola* III, and *N. sphaeroides* MZ–C4 was grouped, with strong LB and PP support, with the strain *N. sphaeroides* HBHF0604. *N. commune* MZ–C24 was closely related to the strains *N. commune* EV1-KK1 clone 2 and WY1KK1. Phylogenetic analyses showed that the strain *Desmonostoc caucasicum* S. Maltseva, Kulikovskiy et Maltsev MZ–154 formed an independent lineage within the *Desmonostoc* clade.

### 2.2. Strain Growth

**Figure 2:**
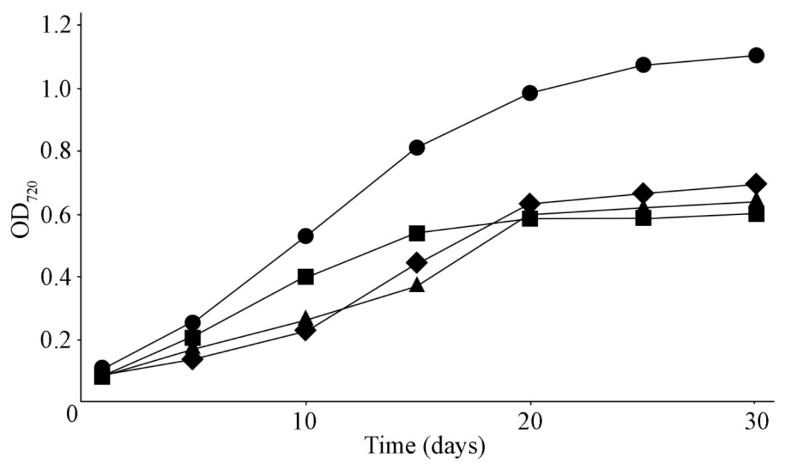
Growth curves of the cyanobacterial strains. Designations: rhombus—*Nostoc sphaeroides* MZ–C4; triangle—*Nostoc calcicola* MZ–C23; rectangle—*Nostoc commune* MZ–C24; circle—*Desmonostoc caucasicum* MZ–C154.

The initial cultures had optical density OD_720_ in the range of 0.082–0.094. The strains *N. sphaeroides* MZ–C4, *N. calcicola* MZ–C23, and *N. commune* MZ–C24 achieved stationary growth on day 20, and *D. caucasicum* MZ–154 on day 25 of cultivation (Figure 2). The dry-mass density by the end of cultivation reached 0.71 ± 0.06, 0.68 ± 0.04, 0.61 ± 0.02, and 1.21 ± 0.13 mg mL^−1^ for MZ–C4, MZ–C23, MZ–C24, and MZ–C154, respectively. The specific growth rate in the logarithmic phase for strains MZ–C4, MZ–C23, and MZ–C24 ranged from 0.026 to 0.028 day^−1^, and for MZ–C154, was 0.41 day^−1^.

### 2.3. Fatty Acid Profiles

The FA profiles were evaluated on the 30th day of growth (stationary phase), and the composition and content of FAs (as % of the total amount) are presented in Table 1.

In strains MZ–C4, MZ–C24, and MZ–C154, saturated FAs dominated and ranged from 96.36 to 98.43%. Monounsaturated (MUFAs) and polyunsaturated fatty acids (PUFAs) ranged from 0.72 to 2.09%, with stearic 18:0 acid as the most common. The content of short-chain FAs was insignificant. It did not exceed 3.13–3.37%, except for the strain *N. commune* MZ–C24, where they amounted to 4.6%. In all three strains, myristic 14:0 acid prevailed among short-chain FAs. The strain *N. calcicola* MZ–C23 differed from the other studied strains in terms of PUFA content, with PUFAs accounting for 57.14% of all FAs. Among PUFAs, α-linolenic 18:3n-3 acid accounted for the most significant amount—39.32%. The strain *N. calcicola* MZ–C23 contained omega-6 FAs in the form of linoleic acid at 17.82%. Another feature of this strain was the absence of stearic 18:0 acid in its profile.

### 2.4. Retinol and α-Tocopherol Content

**Figure 3:**
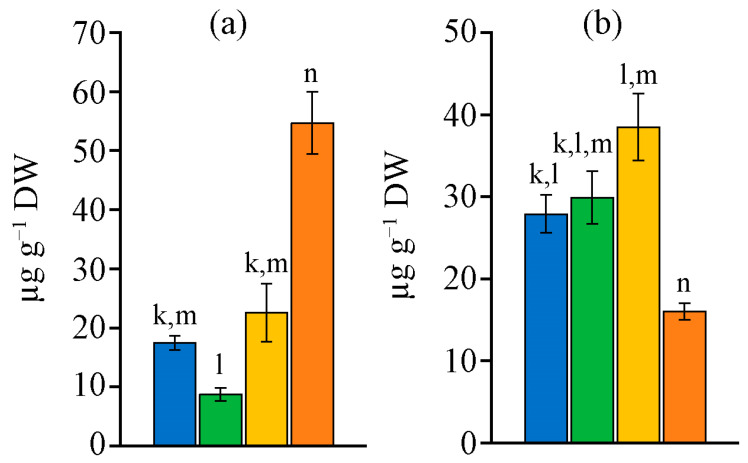
Retinol (**a**) and α-tocopherol (**b**) content in the studied strains (M ± SD, *n* = 5). Note: the letters above the columns indicate unreliable differences relative to the parameters encrypted with the letters. Differences are significant at *p* ≤ 0.05. Colours (and letters above the bars) correspond to the data for blue (k)—*Nostoc sphaeroides* MZ–C4; green (l)—*Nostoc calcicola* MZ–C23; yellow (m)—*Nostoc commune* MZ–C24; and orange (n)—*Desmonostoc caucasicum* MZ–C154.

The retinol content in the biomass of the studied strains varied over a wide range (Figure 3a). The minimum amount of retinol, 8.7 µg g^−1^ dry weight (DW), was found in *N. calcicola* MZ–C23. The biomass of the strains *N. sphaeroides* MZ–C4 and *N. commune* MZ–C24 was richer in retinol. The amount of retinol in their biomass was 2–2.6 times higher than that of MZ–C23. The biomass of strain *D. caucasicum* MZ–C154 was characterised by the highest retinol content and the lowest α-tocopherol (Figure 3b). The highest and lowest (in *N. calcicola* MZ–C23) retinol content differed by 6.3 times. *N. commune* MZ–C24 had the highest amount of α-tocopherol. This value was 2.4 times higher than that of *D. caucasicum* MZ–C154. The remaining two strains did not significantly differ in terms of the amount of α-tocopherol, which on average lay in the range of 27.9–29.9 µg g^−1^ DW.

### 2.5. Antioxidant Defence System Characteristic

**Figure 4:**
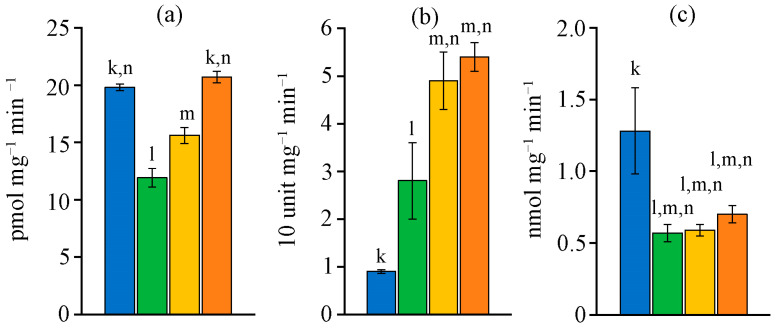
CAT- (**a**), SOD- (**b**), and GPx-activity (**c**) in the studied strains (M ± SD, *n* = 5). Note: the letters above the columns indicate unreliable differences relative to the parameters encrypted with the letters. Differences are significant at *p* ≤ 0.05. Colours (and letters above the bars) correspond to the data for blue (k)—*Nostoc sphaeroides* MZ–C4; green (l)—*Nostoc calcicola* MZ–C23; yellow (m)—*Nostoc commune* MZ–C24; and orange (n)—*Desmonostoc caucasicum* MZ–C154.

CAT-activity did not differ between the studied strains (Figure 4a). The SOD-activity in *D. caucasicum* MZ–C154 and *N. commune* MZ–C24 was almost 2 times higher than in *N. calcicola* MZ–C23, and nearly 5 times higher than in *N. sphaeroides* MZ–C4 (Figure 4b). At the same time, GPx-activity for *N. sphaeroides* MZ–C4 was maximal (Figure 4c).

**Figure 5:**
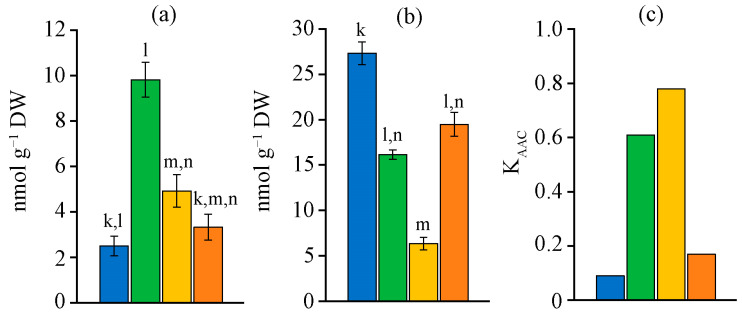
TBARS (**a**), TBARS_in_ (**b**), and K_AAC_ (**c**) in the studied strains (M ± SD, *n* = 5). Note: the letters above the columns indicate unreliable differences relative to the parameters encrypted with the letters. Differences are significant at *p* ≤ 0.05. Colours (and letters above the bars) correspond to the data for blue (k)—*Nostoc sphaeroides* MZ–C4; green (l)—*Nostoc calcicola* MZ–C23; yellow (m)—*Nostoc commune* MZ–C24; and orange (n)—*Desmonostoc caucasicum* MZ–C154.

The intensity of peroxide oxidation processes was maximal in *Nostoc calcicola* MZ–C23 and exceeded the minimum in *Nostoc sphaeroides* MZ–C4 by 3.9 times; the TBARS content confirms this (Figure 5a).

After the initiation of peroxidation, the content of TBA-reactive substances increased in all strains, but to varying degrees. TBARS_in_ increased most significantly (by 10.9 times) in *N. sphaeroides* MZ–C4 (Figure 5b). In *D. caucasicum* MZ–C154, this parameter increased by 5.8 times. The content of TBA-reactive substances in the initial biomass and after initiation of lipid peroxidation (LPO) by the Fenton reaction (Fe^2+^–induced LPO) did not significantly differ in *N. commune* MZ–C24.

The indicator of antioxidant activity (K_AAC_) was similar in the pair *N. calcicola* MZ–C23 and *N. commune* MZ–C24 and in the pair *N. sphaeroides* MZ–C4 and *D. caucasicum* MZ–C154 (Figure 5c). This similarity may indicate similar potential for resistance to LPO in these pairs of strains under the studied cultivation conditions.

### 2.6. Succinate Dehydrogenase Activity

**Figure 6:**
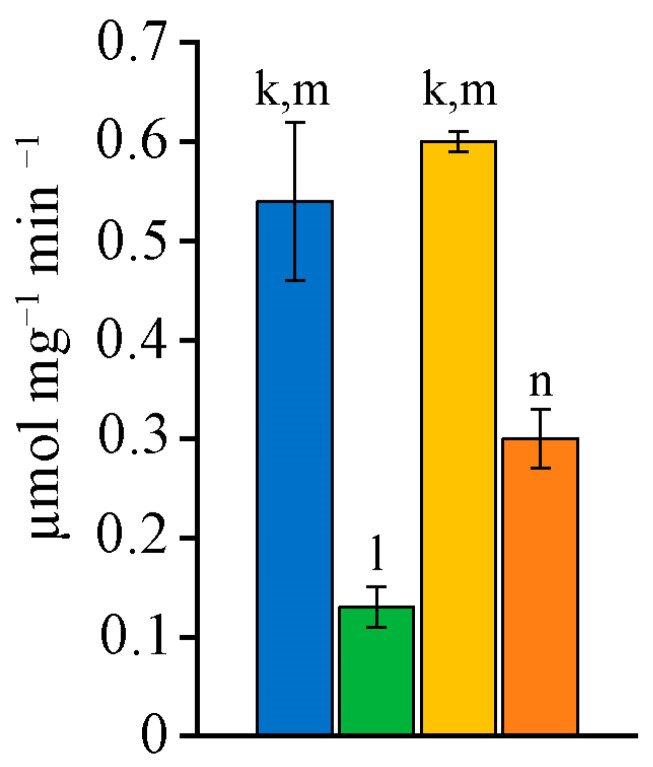
SDH-activity in the studied strains (M ± SD, *n* = 5). Note: the letters above the columns indicate unreliable differences relative to the parameters encrypted with the letters. Differences are significant at *p* ≤ 0.05. Colours (and letters above the bars) correspond to the data for blue (k)—*Nostoc sphaeroides* MZ–C4; green (l)—*Nostoc calcicola* MZ–C23; yellow (m)—*Nostoc commune* MZ–C24; and orange (n)—*Desmonostoc caucasicum* MZ–C154.

The succinate dehydrogenase (SDH) activity was maximal in *N. sphaeroides* MZ–C4 and *N. commune* MZ–C24 and did not differ significantly between the strains (Figure 6). At the same time, the SDH-activity in these strains differed substantially from that in *D. caucasicum* MZ–C154 and *N. calcicola* MZ–C23, which had low SDH-activity. The increased activity of the enzyme indicates an intensive course of anaerobic reactions, which ensure energy processes in strains.

### 2.7. Evaluation of the Relationship of the Measured Parameters in the Studied Strains

To generalise, identify patterns, and identify hidden patterns in changes in the vitamin content and activity of various enzyme groups in the studied strains, we applied principal component analysis (PCA). This method allows for converting a large number of related variables into a smaller number, which facilitates the analysis and interpretation of results. New variables (main components, factors) are a linear combination of the original variables. They describe the maximum amount of variability in the data. The number of principal components (factors) was determined based on the correlation matrix’s own values. Usually, only those factors that have an internal value above one are selected. In addition, each principal component (factor) is characterised by the percentage of explained variation. The PCA demonstrated that the first three PCs are the most significant (eigenvalues > 1) and they explain a total of 97.85% of the total variability of the experimental data (Table 2). An essential step in the analysis is also to identify the variables that form each factor, as well as the absolute values of factor loads for each variable (Table 2). Factor loads of 0.7 or more indicate a powerful relationship between the variable and the factor; values greater than or equal to 0.5 indicate a strong and moderate relationship. When interpreting the results, it is also essential to trace the direction of the relationship: a direct or inverse relationship between a variable and a factor. Consistent use of the above actions allowed us to make several observations.

**Figure 7:**
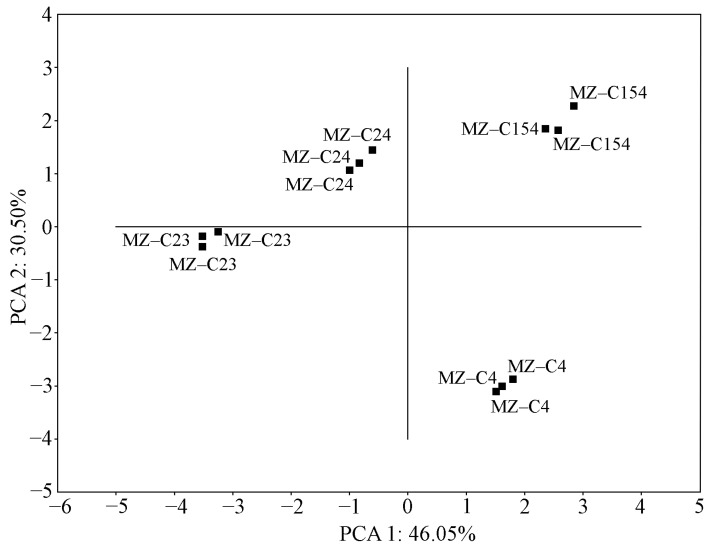
Projection of observations on the PC 1 and PC 2 planes. Designations: MZ–C4—*Nostoc sphaeroides* MZ–C4; MZ–C23—*Nostoc calcicola* MZ–C23; MZ–C24—*Nostoc commune* MZ–C24; MZ–C154—*Desmonostoc caucasicum* MZ–C154.

**Figure 8:**
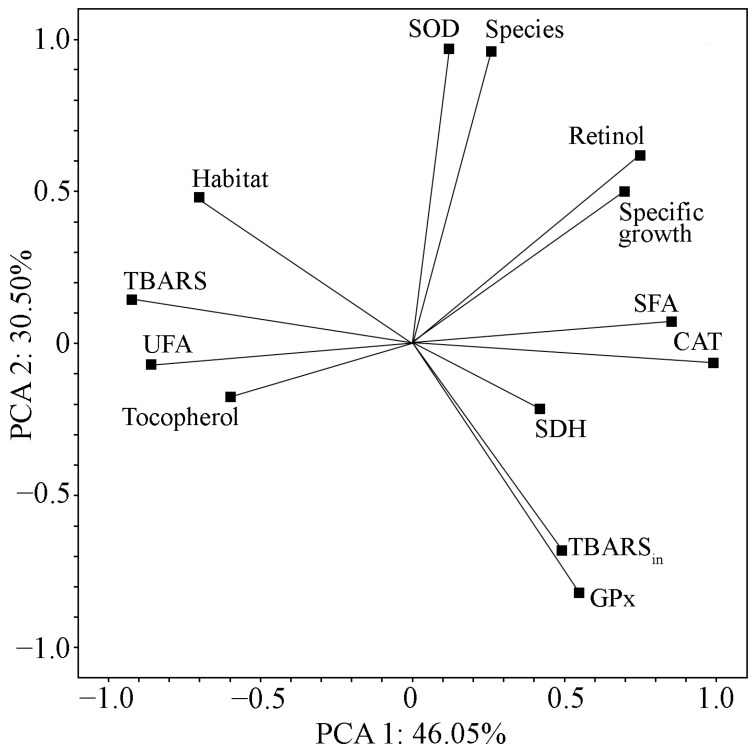
Projection of the studied indicators on the PC 1 and PC 2 planes. Note: Specific growth—specific growth rate; Retinol—retinol content; Tocopherol—α-tocopherol content; CAT—catalase activity; SOD—superoxide dismutase activity; GPx—glutathione peroxidase activity; SDH—succinate dehydrogenase activity; TBARS—TBA-reactive substance concentration in biomass; TBARS_in_—TBA-reactive substance concentration in biomass after LPO induction by Fenton reaction; SFA—relative content of saturated fatty acids in fatty acid profile; UFA—the relative content unsaturated fatty acid in fatty acid profiles; Species—taxonomic affiliation; Habitat—ecological parameters of original habitat.

**Figure 9:**
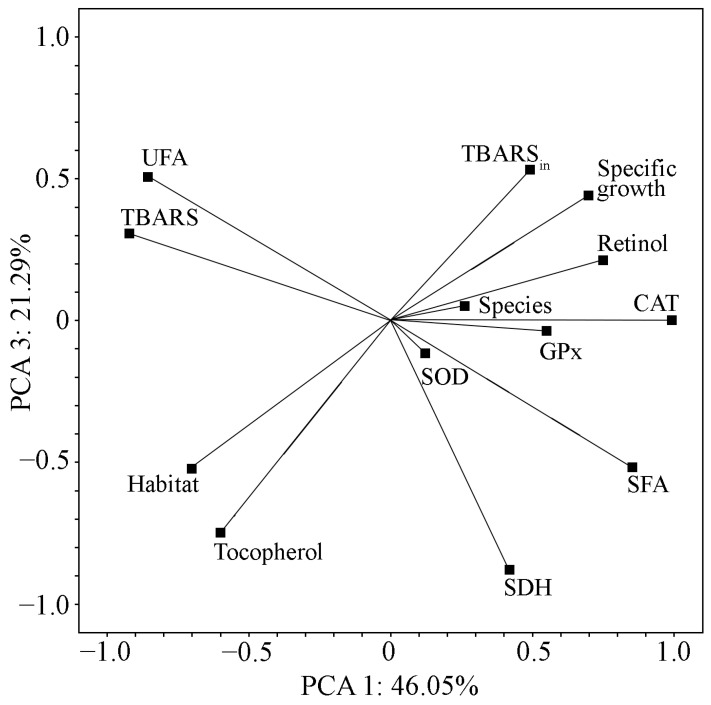
Projection of the studied indicators on the PC 1 and PC 3 planes. Note: Specific growth—specific growth rate; Retinol—retinol content; Tocopherol—α-tocopherol content; CAT—catalase activity; SOD—superoxide dismutase activity; GPx—glutathione peroxidase activity; SDH—succinate dehydrogenase activity; TBARS—TBA-reactive substance concentration in biomass; TBARS_in_—TBA-reactive substance concentration in biomass after LPO induction by Fenton reaction; SFA—relative content of saturated fatty acids in fatty acid profile; UFA—the relative content unsaturated fatty acid in fatty acid profiles; Species—taxonomic affiliation; Habitat—ecological parameters of original habitat.

The results indicate differences among the studied cyanobacterial strains regarding their biochemical and antioxidant properties. The strains formed separate groups on the PCA graph with the projection of observations on PC 1 and PC 2 (Figure 7). Figure 8 and Figure 9 show projections of the studied variables on PC 1 and PC 2 and on PC 1 and PC 3.

At the same time, PC 1 mainly reflects changes in CAT-activity, the content of FAs and TBARS, and retinol, as well as the specific growth rate of strains and their relationships with ecological parameters of the initial habitat (in our study, these are pH, humus content in the soil, and type of phytocenosis). At the same time, CAT-activity, retinol content, and SFA in biomass of the studied strains on the PC 1 and PC 2 planes show an inverse correlation with such parameters as UFA and TBARS. The strongest negative correlations, expressed as the Pearson correlation coefficient (r), are between CAT-activity and both UFA and TBARS, as well as between SFA and both UFA and TBARS (r = −0.84, r = −0.89, r = −0.99, and r = −0.93, respectively). However, the relationships between retinol and both UFA and TBARS are weak: r = −0.58, r = –0.54.

PC 2 had a strong direct relationship with parameters such as SOD-activity and the taxonomic affiliation of strains, and an inverse relationship with GPx-activity and the content of TBARS after initiation of peroxidation (TBARS_in_). The correlation coefficient between SOD- and GPx-activities and TBARS_in_ confirmed a moderate level of association: r = −0.71 and r = −0.65, respectively.

The α-tocopherol content and SDH-activity are closely related to PC 3. The individual coordinates of these parameters are quite far from each other (Figure 9). The correlation between α-tocopherol and SDH-activity is low (r = 0.44).

These results indicate that the cell’s metabolic pathways are dynamically changing towards an increase or decrease in the content and activity of specific components of the AOS, with the help of which the cells of the studied strains cope with stressful conditions. An increase in CAT-activity coincides with an increase in retinol and SFA content in the FA profile. An increase in α-tocopherol coincides with increased SDH-activity, while there is an inverse relationship between SOD- and GPx-activities.

## 3. Discussion

Nitrogen is one of the most essential elements for the growth and development of living organisms. It plays a key role in various physiological and biochemical processes necessary for their viability. In the natural environment, nitrogen, as a source of cyanobacterial growth, can be represented by ammonium (NH_4_^+^), nitrate (NO_3_^−^), nitrite (NO_2_^−^), and urea ((NH_2_)_2_CO) [29]. Nitrogen gas is available to some cyanobacteria [9]. Nitrogen gas fixation in cyanobacteria occurs in specialised cells (heterocytes), with the participation of the enzyme nitrogenase.

Nitrogen starvation in cyanobacteria stimulates the transition from vegetative cells to the formation of heterocytes. At the same time, the expression of genes responsible for the use of N_2_ increases. At the same time, the expression of genes encoding proteins necessary for the growth and division of vegetative cells (basic metabolism) decreases. The genes encoding direct or indirect transcription regulators and signal transducers (NrrA, HetR, PknE, NrrA, HetR) are activated first. The low nitrogen content in the medium first activates NrrA expression, and then NrrA regulates HetR expression, which leads to heterocyte differentiation. Also, HetR, at an early stage of heterocyte differentiation, induces the expression of the PknE gene, subsequently leading to the inhibition of HetR. The genes then activate the biosynthesis of products synthesising the polysaccharide and glycolipid structural components of the heterocyte shell. The genes then activate the synthesis of proteins providing nitrogenase functions, including all nitrogenase proteins [30]. Studies show that the transition of cyanobacteria to the use of N_2_ is slow. During the transition period, cells experience nitrogen deficiency, leading to oxidative stress development. As the results of this study show, the strategy of overcoming it differs among different representatives of cyanobacteria. The effectiveness of protective mechanisms affects the growth rate of cyanobacteria, the composition of FAs, the accumulation of compounds with antioxidant activity, and the activity of enzymes involved in cell AOSs.

It is likely that the cellular mechanisms for maintaining balanced ROS levels under oxidative stress will be specific at the level of different species and strains. Mishra et al. [31] revealed differences in the increased activity of various antioxidant enzymes under oxidative stress caused by Cu2+ and high temperature in a wild and acclimatised strain of *Trichormus doliolum* (Bharadwaja) Komárek et Anagnostidis. Further research will clarify these observations.

The growth rate of cyanobacteria, as well as microalgae, varies widely: *Desmonostoc muscorum* (Bornet et Flahault) Hrouzek et S.Ventura 0.11–0.723 day^−1^ [16]; *Limnospira platensis* (Gomont) K.R.S.Santos et Hentschke 0.06–1.284 day^−1^ [32,33]; *Nostoc* sp. CCIBt3249 0.7–0.8 day^−1^ [34]; *Nostoc* sp. 136 0.01–0.149 day^−1^ [17]; *Oscillatoria* sp. 0.082 day^−1^ [35]; *Chlorella sorokiniana* Shihira et R.W.Krauss 0.081 day^−1^ [36]; and *Chlorella vulgaris* Beijerinck 0.13–1.34 day^−1^ [37]. The growth rate may differ not only between different species, but also between strains of the same species. Deficiency of nutrients, including nitrogen, significantly affects the growth rate. Various studies confirm the significant effect of nitrogen deficiency on growth rate. For example, Toumi and Politaeva [36] observed a 2.2-fold decrease in the growth rate of *Chlorella sorokiniana* when cultured on a medium containing no nitrogen, compared with growth on a medium containing 0.064 g L^−1^ of total nitrogen in the form of NO_3_–N. In *Nostoc* sp. 136, both *Trichormus variabilis* (Kützing ex Bornet et Flahault) Komárek et Anagnostidis and *D. muscorum* reduce growth and productivity in a nutrient medium with a deficiency or complete absence of nitrogen [16,17]. In our experiment, the specific growth rate of *N. sphaeroides* MZ–C4, *N. calcicola* MZ–C23, *N. commune* MZ–C24, and *D. caucasicum* MZ–C154 was 2.6–4.1 times higher than that of cyanobacteria that grew in a cultivation medium with and without nitrogen deficiency [16,17]. The high growth rate in our experiment indicates fairly good resistance of the cyanobacteria studied in this work to oxidative stress during nitrogen starvation.

According to various studies, the FA composition of microalgae and cyanobacteria has a particular specificity at the level of department and class. The FA profile of cyanobacteria contains large amounts of 16:0, 16:1, and PUFAs with 18 carbon atoms: 18:2n-6, 18:3n-3, 18:3n-6, 18:4n-3 [26,38]. The FA profile of all cyanobacteria studied in this work contained 16:0 in an amount of 23.46–27.28%. Palmitoleic 16:1n-7 acid was absent only in *N. commune* MZ–C24. Stearic 18:0 acid prevailed in the composition of the studied cyanobacteria. However, *N. calcicola* MZ–C23 did not contain stearic 18:0 acid. At the same time, the FA profile of *N. calcicola* MZ–C23 contained a large amount of linoleic 18:2n-6 acid and linolenic 18:3n-3 acid. Lang et al. [26] made similar observations for *N. calcicola* SAG 1453-1 and *N. commune* SAG 1453-5. The FA profiles of *N. calcicola* SAG 1453-1 and *N. commune* SAG 1453-5 also lacked stearic 18:0 acid, and the amounts of linoleic 18:2n-6 and linolenic 18:3n-3 acids were high and ranged from 12.37 to 16.80% and 37.1 to 37.68%, respectively (Table 1).

Cyanobacteria and microalgae usually contain small amounts of medium-length FA (C10–C14). Its content generally accounts for less than 1% of all FA [26]. The cyanobacteria studied contained myristic 14:0 and lauric 12:0 acids. Moreover, the latter occurs in minor quantities and only in *N. commune* MZ–C24.

FAs with a very long hydrocarbon chain (C20–C22 and more) are not typical for cyanobacteria. Cyanobacteria usually accumulate these acids in small amounts [26]. For example, *Synechococcus* sp. PCC 7942, on the 20th day of cultivation, contained FAs C20–C22, not exceeding 0.46% of the total FA [39]. In our study, *N. commune* MZ–C24 contained a small amount of arachidic 20:0 acid (Table 1).

Omega-3 and omega-6 FAs are of great interest among FAs. They have a wide range of applications in various fields of human economic activity [7,8]. In our study, *N. calcicola* MZ–C23 had high amounts of omega-3 and omega-6 and exceeded the *N. calcicola* SAG 1453-1 content of 6% found in [26]. At the same time, the ratio of omega-3 and omega-6 between different strains of *N. calcicola* was the same (Table 1).

Stress during cultivation, including nutrient deficiency, leads to changes in the FA composition of cyanobacteria and microalgae [16,40,41]. The FA profiles of *N. sphaeroides* MZ–C4, *N. commune* MZ–C24, and *D. caucasicum* MZ–C154 consisted mainly of SFA. It is known that the double bonds in UFA are the target of ROS under oxidative stress [42]. Our result is consistent with the result of El Shafay et al. [16] in their study on *Trichormus variabilis* and *D. muscorum*. Under conditions of limited nitrogen content in the nutrient medium (50% of the control), these cyanobacteria increased the SFA content by more than 23% (up to 59.91%), the amount of MUFA decreased, and the PUFA content increased slightly. In nitrogen-free cultivation, the main FAs were SFA and MUFA. In experiments with nitrogen restriction or deficiency, *Nannochloropsis* sp. and *Tetradesmus dimorphus* (Turpin) M.J.Wynne significantly accumulated SFA and MUFA [40,41]. The changes in *N. calcicola* MZ–C23 were the opposite. The number of SFAs was small, while MUFA and PUFA predominated. This may indicate individual characteristics of protective reactions to oxidative stress in *N. calcicola* MZ–C23 to better protect MUFA and PUFA from ROS.

As is known, AOS components protect metabolic processes in cells from ROS. Low-molecular-weight antioxidants, including vitamins, can limit the development of chain reactions of oxidative stress. α-Tocopherol (the most bioactive form of vitamin E) and retinol (the most bioactive form of vitamin A) have high antioxidant potential. The antioxidant properties of vitamins A, E, C, and D are due to their ability to non-enzymatically regulate ROS by removing, quenching, inactivating, and terminating radical chain reactions of oxidative stress [43].

As a rule, the content of α-tocopherol in cyanobacteria is low, 93.23–177.23 µg g^−1^ DW [44]. However, the α-tocopherol content in the strains we studied is lower than that in those studied by Mudimu et al. [44] in their study regarding cyanobacteria that do not contain heterocytes, but higher than that of *Anabaena cylindrica* Lemmermann, with a concentration of 4 µg g^−1^ DW [45]. This concentration of α-tocopherol is also higher than in the biomass of an *N. commune* sample stored in dry form for less than one year (7.7 µg g^−1^ DW) and a sample stored at −20 °C for more than 16 years (7.8 µg g^−1^ DW) [46]. At the same time, *N. commune* stored at room temperature for over 9 years did not contain α-tocopherol. The maximum α-tocopherol content in cyanobacteria studied in this work is comparable to similar data for *Chloridella neglecta* (Pascher et Geitler) Pascher and *Vischeria stellata* (Chodat) Pascher, which contained α-tocopherol in the range of 40.81 to 45.17 µg g^−1^ DW [44].

Del Mondo et al. [47] reported that the amount of retinol in cyanobacteria and microalgae varies from 0.01 to 4.28 mg g^−1^ DW. The retinol content in the studied strains corresponds to the minimum values among the known data. However, in their study, Aaronson et al. [45] did not find retinol in *Anabaena cylindrica*. At the same time, cyanobacteria, especially *Limnospira platensis,* can be a rich source of vitamin A precursors, particularly β-carotene [48].

A change in the activity of antioxidant enzymes regulates the level of ROS and LPO products in the cell. The results of this work show that CAT-activity in all studied cyanobacteria was almost at the same level. Such values of CAT-activity indicate a similar level of the processes of conversion of H_2_O_2_ into H_2_O and O_2_. At the same time, the SOD- and GPx-activity differed, which obviously reflects the particular adaptations of each species to counteract oxidative stress. SOD protects against the toxic effects of ROS by catalysing the dismutation of O_2_^·−^ (the addition of two hydrogen ions neutralises two superoxide radicals) into H_2_O_2_ and O_2_. The SOD-activity was higher in *N. commune* MZ–C24 and *D. caucasicum* MZ–C154 than in the other strains. Increased SOD-activity indicates intense inactivation of superoxide. As many scientists have established, an increase in the activity of antioxidant enzymes (SOD, CAT, PO, glutathione reductase (GR), etc.) is characteristic of cyanobacteria and microalgae grown under conditions of various abiotic stress, including nitrogen starvation [21,49,50].

Redox-sensitive cell proteins enable the perception, further transformation, and translation of ROS signals into appropriate cellular responses. ROS directly or indirectly oxidise redox-sensitive proteins through redox-sensitive molecules, for example, thioredoxins (TRX) or glutathione (GSH) [20]. The cellular cascade systems for perceiving ROS and triggering responses are complex. There are direct and reverse interactions during the implementation of responses, such as combining signalling pathways and redirecting them. This follows from the logic of the main task of this system, namely, the maintenance of redox homeostasis of cells.

In this study, *N. sphaeroides* MZ–C4 demonstrated increased GPx-activity. GPx catalyses the reduction of lipid hydroperoxides to alcohols. GPx and CAT are also involved in detoxifying H_2_O_2_ to form water. In this case, GPx uses GSH and TRX as reducing agents [51]. GR, in turn, reduces oxidised glutathione (GSSG) with NADPH(H^+^)’s participation. Zamocky et al. [52] reported that GPx protects cyanobacterial cell membranes from oxidative damage by restoring oxidised UFA, using NADPH as an electron donor. The increase in GPx-activity in *N. sphaeroides* MZ–C4 suggests that it plays a more critical role in resistance to oxidative damage in this strain than in others. We found that *N. sphaeroides* MZ–C4 had the lowest K_AAC_ index, reflecting the overall ability of cells to neutralise ROS. *D. caucasicum* MZ–C154 also had a low K_AAC_ score. This may indicate lower resistance of these cyanobacteria to oxidative stress during prolonged cultivation without nitrogen.

SDH is not a direct component of the AOS, but its activity affects the processes that the AOS regulates. SDH-activity plays a vital role in maintaining the redox balance of cells by enabling ATP synthesis through the respiratory chain. Also in the tricarboxylic acid cycle, SDH catalyses the oxidation of succinate to fumarate, reducing ubiquinone (coenzyme Q_10_) to ubiquinol (reduced form of Q_10_). The antioxidant effect of ubiquinol is associated with the reaction of neutralising free radicals. In addition, coenzyme Q_10_ restores the antioxidant activity of α-tocopherol. In our study, PCA reflected the relationship between SDH-activity and the amount of α-tocopherol in PC 3. Their low correlation coefficient reflects the functional specificity of SDH and α-tocopherol. The activity of enzymes involved in electron transfer across the membrane (NADH-cytochrome b5-reductase, NADH/NADPH-oxidoreductase, and NADPH-coenzyme Q-reductase) depends on the concentration of ubiquinol and α-tocopherol. These enzymes together form the redox system of the plasma membrane and ensure the normal functioning of the redox system of the cell [53]. The SDH-activity among the studied strains was higher in *N. sphaeroides* MZ–C4 and *N. commune* MZ–C24. Increased SDH-activity may indicate the need for the formation of more ATP, which is necessary for maintaining the energy required for metabolism and the neutralisation of ROS, including through the use of more energy-consuming mechanisms.

The results of our and other studies indicate a well-formed and regulated system for protecting metabolic processes from oxidative damage in cyanobacteria. This is a reflection of the long history of cyanobacteria adapting to various habitats and building multiple metabolic pathways to protect cells from the damaging effects of stressors and the development of oxidative stress. Regulation of the reducing and oxidative (redox) states is crucial for cell viability. The AOS ensures the maintenance of redox balance [54,55].

The accumulation of non-enzymatic ROS scavengers, their various biochemical properties and localisation, and differential inducibility at the level of transcripts or proteins of antioxidant enzymes make AOSs very flexible units. At the same time, the AOS can control ROS accumulation in time and space [20].

The metabolic activity of AOS components results from a complex interaction system between signalling and regulatory mechanisms. The expression of the corresponding genes and the biosynthesis of proteins and metabolites involved in protective reactions ensure these interactions. Omics research contributes to identifying and understanding the survival mechanisms of cyanobacteria and their resistance to stress [14]. Thus, genomics provides insight into gene expression patterns and regulatory elements. Transcriptomics includes information about the dynamics of expression of these genes under various conditions and explains the complex regulatory networks that control the pathways of antioxidant protection. Proteomics data helps identify peptide molecules and their modifications. The multiomics approach allows us to evaluate current processes at the system level [10].

The results of molecular interactions and rearrangement of biochemical pathways during the cell’s response to stress can help us assess the indicators of the functional activity of the AOS. These indicators represent the dynamics of changes in the content of low-molecular-weight (non-enzymatic) components of the AOS in cells and the activity of certain groups of antioxidant enzymes. The growth rate of cyanobacteria and changes in the amount and ratio of SFA and UFA can also be marker indicators. Changes in the specific growth rate or biomass productivity also characterise the stress resistance of individual species or strains of cyanobacteria. In general, these indicators can form the basis of various models of redox homeostasis of cyanobacterial cells. Researchers can use these indicators to develop a metabolic interface to describe the signals the cell receives from the environment and implement these signals at various metabolism and AOS functioning levels. Future research will allow us not only to increase the amount of information in this area, but also to study in more depth the metabolic rearrangements in cyanobacteria and the system of protection against oxidative damage.

## 4. Material and Methods

### 4.1. Cyanobacterial Material

The strains *N. sphaeroides* MZ–C4, *N. calcicola* MZ–C23, *N. commune* MZ–C24, and *D. caucasicum* MZ–C154 of cyanobacteria were used in this work. The strains are stored in the Algae Collection of Melitopol State University, CAMU (WDCM 1158) of Melitopol State University (Melitopol) and the Algabank Collection of Cultures and Barcodes of Microalgae and Cyanobacteria (WDCM 1318) of the Timiryazev Institute of Plant Physiology of the Russian Academy of Sciences (Moscow, Russia) as perpetually transferred pure cultures. The strain *N. sphaeroides* MZ–C4 was isolated from a 5 cm soil layer (pH 5.42, humus 8.53%) in a *Juniperus virginiana* L. plantation in the Staro–Berdyansk forest (46°56.11′ N, 35°28.31′ E). The strain *N. calcicola* MZ–C23 was collected from a 5 cm soil layer (pH 6.15, humus 8.18%) in a *Quercus robur* L. plantation of the floodplain of the Staro–Berdyansk forest (46°54.23′ N, 35°29.57′ E). The strain *N. commune* MZ–C24 was isolated from a 5 cm soil layer (pH 6.65, humus 4.19%) in a *Robinia pseudoacacia* L. plantation of the Altagir forest (46°37.16′ N, 35°17.08′ E). The strain *D. caucasicum* MZ–C154 was isolated from mountain meadow subalpine soil (pH 5.6, humus 5.42%) of the Greater Caucasus (43°44.59′ N, 42°40.91′ E) [56].

Identification of the strains MZ–C4, MZ–C23, and MZ–C24 was carried out based on morphological characteristics and 16S rDNA phylogeny. The description of the new species *D*. *caucasicum* was based on morphological characteristics and phylogenetic analysis using the 16S rRNA gene and secondary structure of the ITS region [56].

### 4.2. DNA Extraction, PCR Amplification and Sequencing

Total DNA from the studied strains MZ–C4, MZ–C23, MZ–C2, and MZ–C154 was extracted using Chelex 100 Chelating Resin of molecular-biology grade (Bio-Rad Laboratories, Hercules, CA, USA). The 16S rDNA (total length 1457–1492 bp) was amplified using the pair of primers 8F [57] and B23SR [58]. Amplifications were carried out using PCR mastermixes (ScreenMix, Evrogen, Moscow, Russia). The amplification conditions were as follows: initial denaturation for 5 min at 95 °C followed by 35 cycles of 1 min denaturation at 94 °C, 45 s annealing at 58 °C, and 1 min 40 s extension at 72 °C, with a final extension for 10 min at 72 °C.

PCR products were visualised by horizontal electrophoresis in 1.0% agarose gel stained with SYBR^TM^ Safe (Life Technologies, Carlsbad, CA, USA). The products were purified with a mixture of FastAP, 10×FastAP Buffer, Exonuclease I (Thermo Fisher Scientific, Waltham, MA, USA) and water. The sequencing was performed using a Genetic Analyzer 3500 instrument (Applied Biosystems, Waltham, MA, USA). For sequencing the 16S rDNA, we used the internal primers CYA359F [59] and Primer 8 [60].

Editing and assembling of the consensus sequences were carried out by processing the direct and reverse chromatograms in Ridom TraceEdit ver. 1.1.0 and Mega ver. 7 software [61]. The nucleotide sequences of the 16S rRNA gene were aligned using Mafft ver. 7 software and the G-INS-i model [62]. The reads were included in the alignments along with corresponding sequences of 169 cyanobacteria downloaded from GenBank (names of taxa and Accession Numbers are given in Table S1). The outgroup for the 16S rRNA gene tree comprised two strains of *Rivularia atra* Roth ex Bornet et Flahault (Rivulariaceae, Nostocales). The resulting alignment of the 16S rRNA gene had a length of 1607 characters.

The data set was analysed using the Bayesian inference method implemented in Beast ver. 1.10.1 software [63] to construct a phylogeny. For the alignment partition the most appropriate substitution model, shape parameter α, and a proportion of invariable sites (pinvar) were estimated using the Bayesian information criterion (BIC), as implemented in jModelTest ver. 2.1.10 [64]. This BIC-based model selection procedure selected the GTR + I + G model, pinvar = 0.4610, and α = 0.4130. A Yule process tree prior was used as a speciation model. The analysis ran for 5 million generations with chain sampling every 1000 generations. The parameters-estimated convergence, effective sample size (ESS), and burn-in period were checked using Tracer ver. 1.7.1 software [63]. The initial 25% of the trees were removed and the rest were retained to reconstruct a final phylogeny. The phylogenetic tree and posterior probabilities of branches were obtained based on the remaining trees, which had stable estimates of the parameter models of nucleotide substitutions and likelihood. Maximum Likelihood analysis was performed using RAxML ver. 7.0.4 software [65]. Nonparametric bootstrap analysis with 1000 replicas was conducted. FigTree ver. 1.4.4 software and Adobe Photoshop CC ver. 19.0 were used for viewing and editing the tree.

### 4.3. Growth Assessment

The strain cultivation parameters were selected according to the previously obtained results of studying the physiological processes of cyanobacteria [17,66,67]. At the preparatory stage, strains MZ–C4, MZ–C23, MZ–C24, and MZ–C154 were grown in Z8 medium [68] without the addition of nitrogen (Z8–N) to the exponential phase. A nitrogen-free Z8 medium (Z8–N) was used, since heterocytes may be absent optionally in several cyanobacterial species in media with a high nitrogen content [69]. Then these cultures were used to set up an experiment. For the biochemical analysis, strain MZ–C4, MZ–C23, MZ–C24, and MZ–C154 cultures were maintained in 250 mL Erlenmeyer glass flasks containing 150 mL of Z8–N medium. Analyses of biochemical characteristics and antioxidant potential were carried out according to the scheme presented in our previous studies [70,71,72].

The strains were cultivated using constant orbital shaking (150 rpm in ELMI Sky Line Shaker S-3 L, ELMI Ltd., Riga, Latvia) for 30 days. The cultures grew under standardised conditions, including a temperature of 24 °C, light intensity of 70 μmol photons m^−2^ s^−1^ with a colour temperature of 4000 K, and a 16:8 h light/dark photoperiod. The light intensity and colour temperature were measured using a Sekonic C-800 spectrometer (Sekonic Corporation, Tokyo, Japan). During strain growth, biomass increase was monitored based on optical density changes using an IMPLEN Nanophotometer P300 (Implen GmbH, München, Germany) at λ = 720 nm.

The specific growth rate (μ) was calculated using Equation (1) [73]:

µ = ln (OD_1_ − OD_0_) (t_1_ − t_0_)^−1^ (1)

where OD_1_ and OD_0_ are the optical density on day 1 (t_1_) and day 0 (t_0_), respectively.

### 4.4. Biochemical Parameters

#### 4.4.1. Vitamin A (Retinol) and E (α-Tocopherol) Content Measurement

To prepare a sample, 30 mg of cyanobacterial biomass was separated from the medium by centrifugation (10 min at 3000 rpm). Biomass was previously saponified at 80–85 °C in a KOH solution in ethanol with the addition of 20 mg of ascorbic acid (an antioxidant). α-Tocopherol was isolated from the resulting hydrolysate by sequential extraction with diethyl ether in 2, 1, and 1 mL volumes. The essential extract was washed with water until a neutral reaction occurred on universal indicator paper. Next, the extract was dried by freezing at −18 °C, and filtered through a membrane filter with a pore size of 0.22 µm. The resulting extract was evaporated in a vacuum at 45–55 °C and dissolved in 1 mL of methanol for analysis.

Reverse-phase HPLC determined the content of a-tocopherol in biomass. Determination of α-tocopherol was carried out on a Chromatron-1411 liquid chromatograph (JSC Labtech, Moscow, Russia) with a spectrophotometric UV/VID detector. α-Tocopherol detection was performed at a 292 nm wavelength. An Inspire C18 column (5 µm, 150 mm × 4.6 mm) was used to carry the stationary phase. A mixture of methanol and water was used as the mobile phase in the ratio 96:4 (*v*/*v*), containing 2.5 mM acetic acid/sodium acetate [74], and the mixture was added at a rate of 1 mL min^−1^. The volume of the analysed extract was 10 µL. The separation time was 20 min. During separation, the column temperature was 30 °C. The retention time of α-tocopherol was determined by the retention time of a standard sample from a Merck kit (Millipore, Burlington, MA, USA). For the quantitative determination of α-tocopherol, the calibration curve method was used, which was constructed based on the height and area of peaks, which were obtained by chromatography of calibration solutions with a final concentration of 10–100 µg mL^−1^. Calibration solutions were prepared by diluting a standard solution obtained by diluting α-tocopherol from a Merck kit (Millipore, Burlington, MA, USA) in methanol, with an initial concentration of 1 mg mL^−1^. The concentration was expressed in µg per g of dry weight.

The retinol content was determined by thin-layer chromatography. TLC 60 F254 silica gel thin-layer chromatography plates (Merck, Rahway, NJ, USA) were used as the carrier of the stationary phase. A mixture of n-hexane-diethyl ether in a 9:1 ratio by volume was used as the mobile phase [75]. The volume of the analysed extract was 10 µL. After chromatography, the chromatographic plate was immersed for 5 s in a 1% phosphoric acid solution in ethanol, followed by heating to 110–120 °C in a stream of hot air. After heating, retinol appears as blue spots on a yellow background. The location of the retinol spots on the plate was determined by comparison with the Rf of a standard retinol solution (Sigma-Aldrich, St. Louis, MO, USA). The yellow background was removed through exposure in a chamber saturated with ammonia vapour. To calculate the retinol content, the plates were scanned using a Canon MF 3010 scanner and processed using Sorbfil TLC Videodensitometer v2.3.0 software. The height and area of the peaks corresponding to the retinol spots on the plate were compared with the data of a calibration graph constructed using retinol calibration solutions (Sigma-Aldrich, USA) with a final concentration of 10–100 µg mL^−1^. The concentration was expressed in µg g^−1^ DW.

#### 4.4.2. Succinate Dehydrogenase and Antioxidant Enzyme Activity Measurement

To analyse the enzyme activity, we previously prepared an enzyme extract. To achieve this, 0.1 g of biomass was separated from the medium by centrifugation (10 min at 3000 rpm), then 0.9 mL of phosphate buffer (0.01 M; pH = 7.4) was added to the biomass and homogenised for 30 s using a JY92-IIN ultrasonic homogenizer (Scientz Biotechnology, Ningbo, China) (horn diameter, 6 mm; power, 65 W; frequency, 25 kHz) at a constant temperature of 2–4 °C. The resulting homogenate was centrifuged for 10 min at 10,000 rpm, and the resulting supernatant was used to analyse enzyme activity on the same day.

Succinate dehydrogenase (EC 1.3.99.1) activity was determined using the method of Munujos et al. [76] with minor adaptations. The reaction medium contained 0.5 mM EDTA, 2 mM KCN, 2 mM iodonitrotetrazolium chloride, and 20 mM succinate dissolved in 2 mL phosphate buffer (0.01 M; pH = 7.4). The reaction was started by adding 0.1 mL of the supernatant and incubating for 30 min at 30 °C. Next, using a ULAB 102 spectrophotometer (ULAB, Jinan, China), we measured the optical density at λ = 500 nm (OD_500_), corresponding to the maximum absorption of the coloured formazan iodonitrotetrazolium. The concentration of the formed formazan for activity recalculation was determined using the molar extinction coefficient (19,300).

The CAT-activity (EC 1.11.1.6) was determined using the Hamza and Hadwan [77] method with modification. The volume of the reaction medium was 3 mL. To start the reaction, 0.05 mL of the supernatant was added to 2 mL of hydrogen peroxide (10 mM) and incubated for 10 min at 30 °C. After 10 min, 1 mL of the working reagent (0.04 M aniline sulphate, 0.125 M hydroquinone, 0.04 M ammonium molybdate) was added to the medium and kept at room temperature for 10 min, and the spectrophotometric measurement was taken at λ = 550 nm (OD_550_) using a ULAB 102 spectrophotometer (ULAB, China). The calibration curve of the interaction of hydrogen peroxide in known concentrations with the working solution determined the concentration of reacted hydrogen peroxide to express activity.

GPx-activity (EC 1.11.1.9) was determined by the Sattar et al. [78] method with some modifications. The reaction medium contained 1.5 mL of phosphate buffer (0.01 M; pH = 7.4), 0.2 mL of reduced glutathione (2 mM), and 0.05 mL of a supernatant. The reaction was started by adding 0.2 mL of hydrogen peroxide (2.1 mM) and incubating for 60 min. After that, we added 3 mL of sodium hydrophosphate and 1 mL of Elman’s reagent and we kept the samples for 10 min at 37 °C. The optical density of the solution was measured at λ = 412 nm (OD_412_) using a ULAB 102 spectrophotometer (ULAB, China). To calculate the concentration of reacted glutathione and express enzyme activity, the molar extinction coefficient of the glutathione complex with Elman reagent was used [78].

SOD-activity (EC 1.15.1.1) was determined using the Sirota [79] method. To determine the enzyme activity, we prepared a reaction mixture that contained 2 mL of carbonate buffer (0.2 M; pH = 10.5), nitroblue tetrazolium (50 µM), and 0.05 mL of a supernatant. The reaction was started by adding epinephrine chloride (0.23 mM) to the incubation medium and incubating for 3 min at 25 °C. The optical density of the solution was measured at λ = 560 nm (OD_560_) before and 3 min after adding adrenaline chloride. For measurement, we used a ULAB 102 spectrophotometer (ULAB, China). The activity was calculated using the equation by Sirota [79].

#### 4.4.3. Fatty Acid Analysis, Protein Content, and Thiobarbituric-Acid-Reactive Substance Content

Fatty acid profile analysis was performed using gas-chromatography according to Maltseva et al. [80]. Protein content and thiobarbituric-acid-reactive substance (TBA-reactive substance) content in homogenate (TBARS) and after lipid peroxidation initiation (TBARS_in_) were measured according to Maltseva et al. [72], as was the calculation of the coefficient of antioxidant activity (K_AAC_).

Biomass preparation for the extraction of thiobarbituric-acid-reactive substances was carried out with a 1.2% potassium chloride solution (PanReac AppliChem, Barcelona, Spain). For this purpose, 0.9 mL of potassium chloride was added to 0.1 g of cyanobacterial biomass, which was separated from the medium by centrifugation (10 min at 3000 rpm). Next, biomass was homogenised with an ultrasonic homogeniser JY92-IIN (Scientz Biotechnology, China) with a horn diameter of 6 mm, power of 65 W, and frequency of 25 kHz. The homogenate was centrifuged to separate the supernatant from the precipitate (15 min at 9000 rpm). Supernatant was used for TBARS and TBARS_in_ content determination [72].

### 4.5. Data Analysis

All measurements were carried out in 3–5 repetitions. The graphs show the average values and standard deviation. Statistical analysis was carried out using XLSTAT 2018 software (New York, NY, USA). Statistics were obtained in Microsoft Excel ver. 1903 software (Microsoft Office, Redmond, WA, USA) using single-factor dispersion analysis (ANOVA). Differences at *p* ≤ 0.05 were considered reliable [81].

To generalise and identify patterns in changes in the vitamin content and activity of various enzyme groups in the studied strains, principal component analysis was applied using the Statistica 12.0 program. This method simplifies the model system, better describes a complex nonlinear system of relationships, and dynamically evaluates the system’s initial variables [72].

## 5. Conclusions

The work shows that the *Nostoc* and *Desmonostoc* strains have an effective natural antioxidant protection system in nitrogen stress conditions. When cultured on nitrogen-free media, *N. sphaeroides* MZ–C4, *N. calcicola* MZ–C23, *N. commune* MZ–C24, and *D. caucasicum* MZ–C154 remained viable and grew at a level exceeding 2.6–4.1 times that of other cyanobacteria. The scenario of antioxidant protection in the studied cyanobacteria species under the same stress conditions was different. The most significant differences were in the indicators of SOD- and GPx-activities, as well as the content of retinol, α-tocopherol, and TBARS. Differences in the amount of SFA and UFA in the FA profile indicated a different vulnerability of lipids to peroxidation in the studied cyanobacteria.

The change in SDH-activity reflected not only the dynamics of energy metabolism, but also possible involvement, on the one hand, in increasing ROS production, and on the other, in the activation of α-tocopherol and protection from oxidative damage due to its binding to coenzyme Q10. This mainly reflects the complex nature of interactions in the redox systems of cyanobacteria.

Along with the variability of the reaction of the studied cyanobacteria to nitrogen starvation, certain regular events were noted: an increase in CAT-activity coincided with an increase in retinol content and the amount of SFA in the FA profile, and an increase in α-tocopherol content coincided with an increase in SDH-activity. At the same time, the relationship between SOD- and GPx-activity was the opposite.

## Figures and Tables

**Table 1:** Fatty acid composition of *Nostoc sphaeroides* MZ–C4, *Nostoc calcicola* MZ–C23, *Nostoc commune* MZ–C24, and *Desmonostoc caucasicum* MZ–C154 strains and related strains.

Fatty Acid	Strain	*N. sphaeroides* MZ–C4 ^a^	*N. calcicola* MZ–C23 ^a^	*N. commune* MZ–C24 ^a^	*D. caucasicum* MZ–C154 ^a^	*N. calcicola* SAG 1453–1	*N. commune* SAG 1453–5	*N. commune*	*D. muscorum* UTEX 389	*D. muscorum* SAG 57.79
	growth phase	stationary	stationary	stationary	stationary	stationary	stationary	stationary	stationary	stationary
	medium	Z8-N	Z8-N	Z8-N	Z8-N	ES Ag	ES Ag	BG-11	BG-11	ES Ag
	references	Present study	Present study	Present study	Present study	[26]	[26]	[27]	[28]	[26]
12:0				0.49 ± 0.03						
14:0		3.37 ± 0.16		3.58 ± 0.05	3.13 ± 0.04				0.92	
14:1								0.3		
15:0				0.6 ± 0.01						
16:0		23.7 ± 0.84	27.28 ± 0.75	23.46 ± 0.73	24.3 ± 0.66	25.3	24.97		15.69	19.74
18:0		69.83 ± 1.16		67.58 ± 1.34	71.0 ± 1.43				8.34	2.49
16:1 ^b^									27.87	
16:1n-7		1.4 ± 0.04	8.57 ± 0.11		0.85 ± 0.01	7.6	23.84	5.1		19.41
16:1n-11								2.9		
16:1n-9								3.6		
16:1n-5								0.6		
17:1 ^b^								0.4		
18:1 ^b^									21.80	
18:1n-9			7.01 ± 0.08	0.66 ± 0.03		6.0		11.2		5.75
18:1n-5				0.89 ± 0.02						
18:1n-13								1.3		
18:1n-11								2.6		
16:2n-4										3.47
16:2n-6								2.1		
17:2n-5										3.58
18:2n-6		1.7 ± 0.02	17.82 ± 0.45	2.09 ± 0.04	0.72 ± 0.02	16.8	12.37	14.5	14.09	7.38
18:2n-5								2.1		
18:2n-3								1.1		
16:3n-4										2.17
18:3n-3			39.32 ± 0.88			37.1	37.68	13.5	11.29	26.9
18:3n-6								3.2		
16:4n-3										6.94
20:0				0.65 ± 0.03						
9-octadecanamid						7.2				
dioic fatty acids								1.4		
branched SFA								4.1		
total SFA		96.9 ± 0.95	27.28 ± 0.75	96.36 ± 0.86	98.43 ± 1.15	25.3	24.97	30.0	24.95	22.23
total MUFA		1.4 ± 0.04	15.58 ± 0.09	1.55 ± 0.02	0.85 ± 0.01	13.6	23.84	28.0	49.67	25.16
total PUFA		1.7 ± 0.02	57.14 ± 0.68	2.09 ± 0.02	0.72 ± 0.02	53.9	50.05	36.5	25.38	50.44
total omega-3			39.32 ± 0.72			37.1	37.68	13.5	11.29	29.07
total omega-6		1.7 ± 0.02	17.82 ± 0.11	2.09 ± 0.02	0.72 ± 0.02	16.8	12.37	14.5	14.9	7.38
omega-3/omega-6			2.21			2.21	3.05	0.93	0.76	3.94

^a^ The data are reported as the mean (% of total FAs) ± SD from three independent biological replicates. ^b^ Double band position not determined.

**Table 2:** Matrix of factor loads of the studied variables for cyanobacteria strains.

Variable	PC 1	PC 2	PC 3
Specific growth	0.697	0.499	0.441
Retinol	0.749	0.619	0.214
Tocopherol	−0.599	−0.175	−0.748
SDH	0.417	−0.214	−0.879
GPx	0.549	−0.819	−0.036
CAT	0.989	−0.063	0.002
SOD	0.120	0.968	−0.115
TBARS	−0.921	0.142	0.307
TBARS_in_	0.491	−0.679	0.5320
SFA	0.851	0.072	−0.517
UFA	−0.857	−0.068	0.508
Species	0.260	0.959	0.052
Habitat	−0.703	0.479	−0.524
Total variance %	46.05	30.5	21.29

Note: Specific growth—specific growth rate; Retinol—retinol content; Tocopherol—α-tocopherol content; CAT—catalase activity; SOD—superoxide dismutase activity; GPx—glutathione peroxidase activity; SDH—succinate dehydrogenase activity; TBARS—TBA-reactive substance concentration in biomass; TBARS_in_—TBA-reactive substance concentration in biomass after LPO induction by Fenton reaction; SFA—relative content of saturated fatty acids in fatty acid profile; UFA—the relative content unsaturated fatty acid in fatty acid profiles; Species—taxonomic affiliation; Habitat—ecological parameters of original habitat.

## Data Availability

The original contributions presented in this study are included in the article/Supplementary Materials. Further inquiries can be directed to the corresponding author.

## Supplementary Materials

The supporting information can be downloaded at: https://www.mdpi.com/article/10.3390/ijms262210891/s1.
