# Supplementary material for: Antioxidant Status of Cyanobacteria Strains During Long-Term Cultivation in Nitrogen-Free Media
Source: Int J Mol Sci. 2025 Nov 10;26(22):10891. doi: 10.3390/ijms262210891 (PMC12652501; doi:10.3390/ijms262210891)
Supplement: Supplementary file 1 [file ijms-26-10891-s001.zip › ijms-3923984-supplementary.pdf]

Supplementary Table S1. Taxa and 16S rDNA sequence data used in phylogenetic analysis

taxa	source	the GenBank accession no.	taxa	source	the GenBank accession no.
Aliinostoc catenatum	SA24	MK503792	*Desmonostoc* sp.	De1	AM711534
Aliinostoc catenatum	SA24	NR 172582	*Desmonostoc* sp.	Ds1	HG004579
Aliinostoc magnakinetifex	SA18	MK503791	*Desmonostoc* sp.	HA4255-MV1	MW344111
Aliinostoc morphoplasticum	NOS	KY403996	*Desmonostoc* sp.	OSNI32S01	HG004587
Aliinostoc morphoplasticum	NOS	NR 158066	*Desmonostoc* sp.	PCC 6302	HG004582
Aliinostoc sp.	CENA175	KC695867	*Desmonostoc* sp.	PCC 7422	HG004586
Aliinostoc sp.	CENA511	KX458482	*Desmonostoc* sp.	PCC 8107	HG004583
Aliinostoc sp.	CENA513	KX458483	*Desmonostoc* sp.	PCC 8306	HG004584
Aliinostoc sp.	CENA514	KX458484	*Desmonostoc* sp.	PCC 9230	HG004585
Aliinostoc sp.	CENA524	KX458485	*Desmonostoc* sp.	PCC 9231	AY742452
Aliinostoc sp.	CENA535	KX458486	*Desmonostoc* sp.	SA5	MF770266
Aliinostoc sp.	CENA536	KX458487	*Desmonostoc* sp.	TO1S01	AM711549
Aliinostoc sp.	CENA543	KX458489	*Desmonostoc* sp.	UAM-307	HM623782
Aliinostoc sp.	CENA544	KX458490	*Desmonostoc vinosum*	HA7617-LM4	KF417429
Aliinostoc sp.	CENA548	KX458492	*Goleter apudmare*	HA4340-LM2	KF417425
Aliinostoc sp.	CENA88	GQ259207	*Goleter apudmare*	HA4356-MV2 clone p8i	JN385289
Aliinostoc sp.	HA4241-MV4	MW344116	*Halotia branconii*	CENA186	KC695877
Aliinostoc sp.	PCC 8112	AM711537	*Halotia branconii*	CENA392	KJ843312
Aliinostoc sp.	PCC 8976	AM711525	*Halotia longispora*	CENA184	KC695875
Aliinostoc sp.	SA9	MK503790	*Halotia longispora*	CENA420	KJ843313
Aliinostoc sp.	SA18	MK354276	*Halotia wernerae*	CENA158	KC695852
Aliinostoc sp.	SA24	MK354277	*Halotia wernerae*	CENA160	KC695854
Aliinostoc sp.	SA43	MK503794	*Komarekiella atlantica*	CCIBT 3481	KX638484
Aliinostoc sp.	SA46	MK503795	*Komarekiella atlantica*	CCIBT 3483	KX638487
Anabaena affinis	NIES-40	AF247591	*Minunostoc cylindricum*	CHAB 5843	MH918062
Anabaena augstumalis	SCMIDKE JAHNKE/4a	AJ630458	*Minunostoc cylindricum*	CHAB 5844-1	MH918063
Anabaena cf. *crassa*	1tu27s7	AJ630413	*Mojavia pulchra*	ACCS 017	GU434232
Anabaena cylindrica	PCC 7122 CCAP 1403/2A	HF678516	*Mojavia pulchra*	JT2-VF2	AY577534
Anabaena flos-aquae	0tu33s15	AJ630420	*Nodularia baltica*	BY1	AJ133177
Anabaena kisseleviana	TAC33	AY701557	*Nodularia harveyana*	BECID29	AJ781146
Anabaena lemmermannii	1tu32s11	AJ630424	*Nodularia sphaerocarpa*	HKVV	AJ133183
Anabaena macrospora	PMC9301	AJ293115	*Nostoc* sp.	ATCC53789	AF062638
Anabaena planctonica	NIES-816	AY701548	*Nostoc calcicola*	AM50C	MG641901
Anabaena spiroides	PMC9403	AJ293116	*Nostoc calcicola*	AM50D	MG641902

Anabaenopsis sp.	PCC 9215	AY038033	*Nostoc calcicola*	III	AJ630447
Aphanizomenon issatschenkoi	TAC419	AY196087	*Nostoc calcicola*	SAG 1453-1	KM019926
Aphanizomenon flos-aquae	PCC 7905	AJ133154	*Nostoc carneum*	IAM M-35	AB325906
Calothrix brevissima	IAM M-249	AB074504	*Nostoc* cf. *indistinguendum*	F15-VF1	AY577541
Coleodesmium wrangelii	144-2C	AF334703	*Nostoc* cf. *indistinguendum*	F15-VF12	AY577539
Compactonostoc shennongjiaensis	CHAB 5781	MH598843	*Nostoc* cf. *indistinguendum*	F15-VF4	AY577540
Cyanospira rippkae	PCC 9501	AY038036	*Nostoc* cf. *lichenoides*	JT1-VF3	AY577532
Cylindrospermopsis raciborskii	NIES993	AB115489	*Nostoc* cf. *lichenoides*	JT1-VF7	MH427698
Desikacharya azollae	Kom BAI/1983	AJ630454	*Nostoc* cf. *verrucosum*		AB245144
Desikacharya constricta	SA10	MK354274	*Nostoc commune*	EV1-KK1 clone 2	MH427702
Desikacharya ellipsosporum	CCAP 1453/2	HF678488	*Nostoc commune*	NC3-K1	EU586722
Desikacharya nostocoides	BHU1-PS	MH036167	*Nostoc commune*	type D	AB933329
Desikacharya piscinale	CENA21	AY218832	*Nostoc commune*	WY1KK1	EU586733
Desikacharya soli	BHU2-PS	MH036168	*Nostoc desertorum*	CM1-VF14	MH427691
Desikacharya sp.	HK-01	AB085687	*Nostoc edaphicum*	X	AJ630449
Desikacharya sp.	KNUA003	JF740671	*Nostoc ellipsosporum*	V	AJ630450
Desikacharya sp.	PCC 6720	DQ185240	*Nostoc entophytum*	IAM M-267	AB093490
Desikacharya sp.	PCC 9426	AM711538	*Nostoc flagelliforme*	IMGA0408	EU178143
Desikacharya sp.	SAG 2306	GQ287649	*Nostoc indistinguendum*	CM1-VF10	MH427692
Desikacharya sp.	TH1S01	AM711547	*Nostoc lichenoides*	CNP-AK1 clone B	MH427689
Desikacharya thermotolerans	9C-PS	KX252675	*Nostoc linckia*	IAM M-251	AB074503
Desmonostoc aggregatum	CF06	MW281056	*Nostoc linckia* var. *arvense*	IAM M-30	AB325907
Desmonostoc aggregatum	CF06-2	MZ263179	*Nostoc minutum*	ACSSI	KY283066
Desmonostoc aggregatum	CF06-3	MZ263180	*Nostoc oromo*	ETH. 2.4. M.5 1	MH427659
Desmonostoc caucasicum	MZ–C154	OM044129	*Nostoc paludosum*	BA033	KX423684
Desmonostoc danxiaense	CHAB5868	MH291266	*Nostoc punctiforme*	PCC73102	AF027655
Desmonostoc danxiaense	CHAB5869	MH291267	*Nostoc* sp.	152	AJ133161
Desmonostoc geniculatum	HA4340-LM1 clone 37A	KU161660	*Nostoc* sp.	Cc3	HG004580
Desmonostoc geniculatum	HA4340-LM1 clone 37C	KU161661	*Nostoc* sp.	GSV224	AF062637
Desmonostoc geniculatum	HA4340-LM1 clone 37D	KU161662	*Nostoc* sp.	Lobaria cyanobiont 34	AF506259
Desmonostoc muscorum	CENA18	AY218827	*Nostoc sphaeroides*	HBHF0604	EU178144
Desmonostoc lechangense	CF01	MW281055	*Nostoc sphaeroides*	ACSSI 150	KY887479
Desmonostoc lechangense	CF01	MZ263177	*Nostoc verrucosum*	KU005	AB494996
Desmonostoc lechangense	CF01-3	MZ263178	*Pseudoaliinostoc constrictum*	SA30	MK503793
Desmonostoc magnisporum	AR6 PS	MH497066	*Pseudoaliinostoc sejongens*	ACKU 594	MT000689
Desmonostoc muscorum	I	AJ630451	*Pseudoaliinostoc soli*	ZH1(3)_PS	MH497065
Desmonostoc muscorum	II	AJ630452	*Pseudoaliinostoc* sp.	ACKU 595	MT000690
Desmonostoc muscorum	Lukesova 1/87	AM711523	*Pseudoaliinostoc* sp.	ACKU 596	MT000691
Desmonostoc muscorum	Lukesova 2/91	AM711524	*Pseudoaliinostoc* sp.	PMC 882.14	MT984291

Desmonostoc muscorum	SAG 57.79	KM019934	*Pseudoaliinostoc tiwarii*	LI_PS	MH497064
Desmonostoc persicum	SA14	MF642332	*Rexia erecta*	CAT 1M	AY452533
Desmonostoc punense	MCC 2741	KT166436	*Rivularia atra*	BIR KRIV1	AM230674
Desmonostoc salinum	CCM-UFV059	KX787933	*Rivularia atra*	BIR MGR1	AM230675
Desmonostoc sp.	111 CR4 BG11N	KF761564	*Roholtiella edaphica*	JOH39	KM268878
Desmonostoc sp.	111 CR4 BG11B	KF761565	*Roholtiella edaphica*	RU1	KM268879
Desmonostoc sp.	7N clone NC3C	KF934182	*Roholtiella mojaviensis*	WJT36-NPBG5B	KM268892
Desmonostoc sp.	81 NMI ANAB	KF761562	*Spirirestis rafaelensis*	143-2B	AF334691
Desmonostoc sp.	8938	AY742454	*Tolypothrix distorta*	163-5B	AF334694
Desmonostoc sp.	8964:3	AM711541	*Tolypothrix* sp.	IAM M-259	AB093486
Desmonostoc sp.	CCIBT 3489	KX638490	*Trichormus variabilis*	GREIFSWALD	AJ630457
Desmonostoc sp.	Cc2	AM711532	*Trichormus variabilis*	HINDAK 2001/4	AJ630456
Desmonostoc sp.	Cr3	HG004581	*Violetonostoc minutum*	CHAB5840	MN400069
Desmonostoc sp.	Cr4	AM711533	*Violetonostoc minutum*	CHAB5841	MN400070
